# *Eucommia ulmoides* Oliv. leaves flavonoids attenuate methylglyoxal‐induced endothelial cell apoptosis in vitro and in vivo by upregulating AKT‐Nrf2 signaling and downregulating oxidative stress

**DOI:** 10.1002/fsn3.4416

**Published:** 2024-08-16

**Authors:** Xin Deng, Qianfeng Wu, Youping Liu

**Affiliations:** ^1^ School of Pharmacy Chengdu University of Traditional Chinese Medicine Chengdu China; ^2^ Basic Medicine Research Innovation Center for Cardiometabolic Diseases, Ministry of Education Southwest Medical University Luzhou China

**Keywords:** apoptosis, endothelial, *Eucommia ulmoides* Oliv. leaves, methylglyoxal, ROS

## Abstract

Methylglyoxal (MGO) triggers oxidative stress responses in vascular endothelial cells, leading to apoptosis linked to diabetic vascular complications. Total flavonoids of *Eucommia ulmoides* leaves (TFEL) display antioxidant activity, yet its prevention of MGO‐induced apoptosis and mechanisms are unclear. Our study used western blotting and ELISA to evaluate protein levels and enzyme activities. Cell viability and apoptosis were evaluated using CCK8 assay and PE Annexin V/7‐AAD double staining. Reactive oxygen species (ROS) generation and mitochondrial membrane potential (MMP) were measured using fluorescence probes. Vascular pathological changes and apoptosis were analyzed through H&E and TUNEL staining. In vitro, MGO‐stimulated human umbilical vein endothelial cells (HUVECs) were treated with varying TFEL concentrations. Our results demonstrated that TFEL significantly enhanced cell viability, reduced apoptosis, downregulated caspase‐3 activity, and Bax/Bcl‐2 ratio. Moreover, TFEL markedly suppressed MGO‐induced ROS and malondialdehyde (MDA) production while restoring antioxidant enzyme activity and MMP. TFEL pretreatment promoted the expression of p‐Akt, Nrf2, and HO‐1 proteins. Pharmacological inhibition of p‐Akt significantly suppressed the upregulation of Nrf2 and HO‐1 protein levels mediated by TFEL. Consistently, pharmacological inhibition of Nrf2 or p‐Akt partially abrogated the protective effects of TFEL against MGO‐induced damage in HUVECs. In vivo studies revealed that TFEL (100 and 200 mg/kg) partially restored antioxidant capacity and reduced aortic thickness and apoptosis in MGO‐injured mice. In conclusion, the findings indicate that TFEL mitigates MGO‐induced apoptosis via activation of p‐Akt/Nrf2/HO‐1 and scavenging of oxidative stress, highlighting its potential in diabetic vascular complication management.

## Graphical abstract

A highly active dicarbonyl compound, methylglyoxal's (MGO) formation and accumulation have been associated with the occurrence of diabetic vascular complications, including oxidative stress‐induced endothelial cell apoptosis. This study suggested that total flavonoids of *Eucommia ulmoides* Oliv. leaves (TFEL) alleviated oxidative stress and protected against MGO‐induced vascular endothelial cell apoptosis by activating the AKT‐Nrf2 signaling pathway.

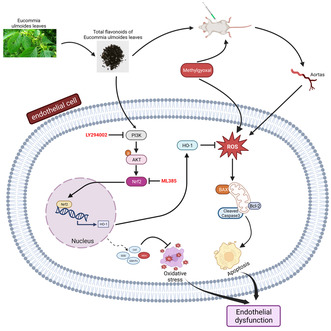

## 1 INTRODUCTION

Diabetes mellitus, a persistent metabolic dysregulation, is defined by the presence of insulin resistance and dysfunctional glucose homeostasis (DeFronzo et al., 2015; Majety et al., 2023). According to Saeedi et al. (2019), the prevalence of diabetes is approximately 425 million globally and is projected to approximately double by 2045. Despite advances in blood glucose management, vascular complications of diabetes have not decreased (Ali et al., 2022). Currently, clinical strategies for diabetes vascular complications mainly focus on preventive treatments. However, the most widely used clinical interventions, such as statins, angiotensin converting enzyme inhibitors, and hypoglycemic therapies, are unfortunately insufficient to prevent the development of vascular complications (Rask‐Madsen & King, 2013; Schalkwijk & Stehouwer, 2020). Endothelial cells (ECs), which form a monolayer cell barrier covering the luminal surface of blood vessels, play a crucial role in regulating vascular morphology, function, and metabolic homeostasis (Michiels, 2003; Trimm & Red‐Horse, 2023). Evidence suggests that endothelial dysfunction‐induced imbalance in the vascular homeostasis is a major contributor to diabetes‐related vascular complications (Otto et al., 2020; Shi & Vanhoutte, 2017). While the exact underlying mechanism for this imbalance is unknown, diabetes‐associated oxidative stress and endothelial cell apoptosis are unique to this disorder (Guo et al., 2023; Hu et al., 2020). Multiple investigations have demonstrated that the elimination of reactive oxygen species (ROS) can notably alleviate endothelial cell death triggered by both in vivo and in vitro diabetic models (Jarisarapurin et al., 2021; Lee et al., 2020; Wang et al., 2022). Consequently, inhibiting endothelial cell apoptosis emerges as a viable strategy for the prevention and amelioration of diabetic vascular complications.

Methylglyoxal (MGO), a highly reactive dicarbonyl metabolite, is primarily generated through non‐enzymatic degradation processes involving glyceraldehyde‐3‐phosphate and dihydroxyacetone‐phosphate (Schalkwijk & Stehouwer, 2020). Accumulation of MGO has been implicated in the development and progression of various diseases, including diabetic complications, obesity, atherosclerosis, degenerative neurological disorders, and cancers (Bellahcène et al., 2018; Chun et al., 2016). The underlying mechanism by which MGO leads to diabetic vascular complications is complex. MGO‐induced endothelial apoptosis has been found as a major pathophysiological foundation for diabetic vascular complications due to disrupted antioxidant defense, culminating in elevated oxidative stress (Jarisarapurin et al., 2021; Phalitakul et al., 2013). Excessive levels of ROS, along with a reduction in mitochondrial membrane potential (MMP) and increased mitochondrial permeability, result in oxidative stress. This, in turn, triggers an apoptotic process that is dependent on caspase cascade activation (Sadek et al., 2017). The nuclear erythroid 2–related factor 2 (Nrf2) transcription factor is pivotal in regulating the antioxidant defense system, thereby safeguarding cells against ROS. Loboda et al. (2016) report that increased Nrf2 levels boost the activity of antioxidant enzymes, including superoxide dismutase 2 (SOD2) and heme oxygenase‐1 (HO‐1). Moreover, the AKT‐mediated Nrf2/HO‐1 signaling pathway has been deemed crucial in managing oxidative stress and apoptosis induced by MGO (Li et al., 2018). Consequently, it emerges as a potential target for the prevention and treatment of diabetic vascular complications.

*Eucommia ulmoides* Oliv is widely utilized in traditional Chinese, Japanese, and Korean medicine. Chinese medical classics record that it nourishes the liver and kidneys, strengthens muscles and bones, and prolongs life (Huang et al., 2021). Modern pharmacological research corroborates these benefits, demonstrating that *E. ulmoides* has lipid‐lowering, antioxidant, anti‐obesity, and blood glucose‐reducing effects (Huang et al., 2021). The leaves of *E. ulmoides* are used to produce Du‐Zhong tea which is beneficial for individuals with diabetes, hypercholesterolemia, fatty liver, and hypertension (Shi et al., 2022). *E. ulmoides* leaves have a similar chemical content to their bark and are enriched in lignans, flavonoids, phenols, iridoids, steroids, and terpenes (Shi et al., 2022). Flavonoids are abundant in the leaves and possess potent antioxidant properties. Park et al. (2006) demonstrated that leaf flavonoids of *E. ulmoides* possess hypoglycemic properties and can amplify endogenous antioxidant activities in a type 2 diabetic mice model. Previous investigations have uncovered protective effects of flavonoid extracts derived from diverse plants on vascular endothelial cell damage. For instance, flavonoid extracts derived from *Piper sarmentosum* can significantly enhance the viability of hydrogen peroxide‐damaged HUVECs (Ugusman et al., 2012); resveratrol combined with total flavones of hawthorn exert protective effects on endothelial cell damage after coronary artery bypass grafting (Zhu et al., 2018); and total flavonoids from *Astragalus* may exert protective effects on hypoxia‐induced HUVECs damage through the Akt/eNOS signaling pathway (Liu et al., 2018). Based on these findings, it is speculated that flavonoids may have protective effects on diabetes‐related endothelial injury.

The present investigation assessed the possible influences of the total flavonoids from *E. ulmoides* (TFEL) on MGO‐induced endothelial cellular injury including apoptosis were examined through the utilization of in vitro and in vivo models. In addition, we also investigated the protective mechanism of TFEL against MGO‐induced oxidative stress and apoptosis in endothelial cells through pharmacological inhibition of Nrf2 and phosphorylated AKT (p‐Akt).

## 2 MATERIALS AND METHODS

### 2.1 Extraction of TFEL

In October 2021, *E. ulmoides* leaves were collected from Luzhou City, Sichuan Province, China. Professor Ji Tian from the School of Pharmacy at Southwest Medical University (located in Luzhou, China) identified the harvested leaves. The extraction method for obtaining TFEL was adapted from a previously published study (Peng et al., 2021). Briefly, dehydrated *E. ulmoides* leaves were pulverized, filtered, and sieved through a 40‐mesh sieve. The powder was suspended in 80% ethanol and ultrasonicated for 30 min and then heated under reflux for two 1 h cycles. The resulting liquid was then filtered and subjected to column chromatography using an AB‐8 macroporous resin (Macklin, Shanghai, China) that was washed with dH_2_O and 10% ethanol and TFEL was eluted with 80% ethanol. Ethanol was eliminated through reduced pressure, and the leftover liquid underwent freeze‐drying to obtain TFEL powder. A total of 10.2 kg of dried *E. ulmoides* leaves were extracted to obtain 229.5 g of TFEL dry powder, with a yield of 2.25%.

### 2.2 High‐performance liquid chromatography (HPLC) analysis

The chemical composition of TFEL was analyzed using HPLC with a BDS Hypersil C18 column (250 mm × 4.6 mm, 5 μM) (Thermo, Pittsburg, PA, USA). The flow rate of the system was set at 1 mL per minute, with an injection volume of 10 μL and a detection wavelength of 360 nm. Mobile phase A was acetonitrile, and mobile phase B was 0.1% phosphoric acid. TFEL were eluted using a gradient elution as follows: 0–10 min, 92%–86% Band 10–50 min, 86%–84% B. A mixed standard solution was prepared using quercetin‐3‐*O*‐β‐d‐glucopyranosyl‐(1 → 2)‐α‐l‐arabinopyranoside, rutin, isoquercitrin, and astragalin to generate a standard curve.

### 2.3 Animal models and TFEL administration procedures

C57BL/6 males aged 6 weeks were purchased from Gempharmatech (Jiangsu, China) and acclimatized for a duration of 1 week in a standardized environment maintained at 22°C with a humidity range of 50%–70%, and a regulated light/dark cycle of precisely 12 h. Thereafter the experimental subjects were sorted into six groups (with *n* = 6 per group) at random: Vehicle, MGO (Sigma‐Aldrich, St. Louis, MO, USA), TFEL (200 mg/kg), MGO + TFEL (50 mg/kg), MGO + TFEL (100 mg/kg), and MGO + TFEL (200 mg/kg). MGO was dissolved in 0.9% saline whereas TFEL was prepared as a suspension in 0.9% saline. MGO was administered intraperitoneally 5 days per week for 7 weeks; 50 mg/kg for weeks 1 and 2, 60 mg/kg for weeks 3 and 4 and 75 mg/kg for weeks 5–7. TFEL was administered intragastrically daily for weeks 3–7. The vehicle group received 0.9% saline injection. Throughout the study, the blood glucose levels, body weights, and food intake were consistently observed and recorded. At the conclusion of the experimentation, blood was gathered, and the abdominal aorta was procured from mice that were sedated using sodium pentobarbital (8 mg/kg, i.p.). The ethics committee of Southwest Medical University for animal experimentation granted approval to the animal study protocol (protocol number: 20221001‐006, date of approval: October 1, 2022).

### 2.4 Cell culture

Human umbilical vein cells (HUVECs) were acquired from ScienCell (Carlsbad, CA, USA), which also provided a culture medium for endothelial cells. This medium was enriched with 5% fetal bovine serum, 1% endothelial cell growth supplement, and 1% antibiotic solution. The HUVECs were subsequently cultivated in a controlled environment of a humidified incubator maintained at 37°C and 5% CO_2_.

### 2.5 Cell viability

The effects of MGO and TFEL on HUVECs viability were evaluated using a cell proliferation assay CCK8 assay (Beyotime Biotechnology, Shanghai, China) using the manufacturer's protocol. In brief, HUVECs were initially seeded in a 96‐well plate at 3 × 10^3^ cells per well and left to incubate for 24 h. Following this, the cells were exposed to TFEL at different concentrations for 2 h prior to MGO stimulation for 24 h. Thereafter, each well received 10 μL of CCK8 solution and was subject to incubation for a duration of 1 h in darkness. Following this, the absorbance was measured at a wavelength of 450 nm using the microplate reader (BioTek, Winooski, VT, USA).

### 2.6 Apoptosis assay

Prior to MGO stimulation for a duration of 24 h, HUVECs were preconditioned with TFEL for a period of 2 h. We investigated the effects of silencing Nrf2 and AKT on TFEL protection against apoptosis by using the cells with ML385 (20 mM) and LY204002 (50 μM) purchased from APExBIO (Houston, TX, USA), which were administered 2 h beforehand MGO treatment. We utilized a commercially available PE Annexin V Apoptosis Detection kit (BD Biosciences, Franklin Lakes, NJ, USA) to conduct an apoptosis assay via flow cytometry, adhering to the manufacturer's guidelines.

### 2.7 Caspase‐3 activity assay

Using a commercially available assay kit from Cell Signaling Technology (Beverly, MA, USA), cell lysates were evaluated for caspase‐3 activity, in accordance with the manufacturer's guidelines. To measure cell fluorescence, a microplate reader (BioTek) was utilized, set to an excitation wavelength of 380 nm and an emission wavelength ranging between 420 and 460 nm.

### 2.8 Detection of intracellular ROS

Cellular ROS concentrations were quantified according to standardized methods by employing a ROS assay kit (Beyotime Biotechnology) incorporating a DCFH‐DA probe to assess intracellular oxidative stress. Briefly, HUVECs were preincubated with TFEL for 2 h before exposure to MGO (200 μM) for 1 h. Subsequently, the cells underwent a washing process using cell culture medium, followed by an incubation with DCFH‐DA (10 μM) for 30 min at 37°C in a dark environment. Subsequently, the cells were washed twice with cell culture medium to eliminate any remaining DCFH‐DA that had not penetrated the cells. The fluorescence intensity was then quantified using a microplate reader (BioTek), with excitation and emission wavelengths set at 488 and 525 nm, respectively. Finally, an inverted fluorescence microscope was utilized to capture the images (Olympus, Tokyo, Japan).

### 2.9 Superoxide dismutase (SOD), catalase (CAT), glutathione peroxidase (GSH‐Px) activity, and malondialdehyde (MDA) content

For assessing oxidative stress, HUVECs were cultivated in 6‐well plates, ensuring the medium was deprived of serum. The medium was refreshed once the cells attained approximately 80% confluence. Subsequently, the cells underwent pretreatment with different TFEL concentrations for a duration of 2 h, followed by stimulation with MGO (200 μM) for 1 h. For the in vivo oxidative stress marker assay, serum was obtained from the collected blood samples of the mice following anesthetization during the final stage of the study. Protein concentrations in cell lysates and serum were quantified by the BCA protein assay. The levels of SOD, CAT, GSH‐Px, and MDA were quantified by utilizing commercial kits (Beyotime Biotechnology) in accordance with the manufacturers' prescribed protocols.

### 2.10 Assessing mitochondrial membrane potential

Mitochondrial membrane potential was assessed using the JC‐1 assay kit (Beyotime Biotechnology) following the manufacturer's instructions. Briefly, HUVECs were subjected to a 2 h pretreatment with TFEL, followed by a 1 h stimulation with MGO at a concentration of 200 μM. The cells were treated with JC‐1 staining solution and subjected to incubation at 37°C for 20 min. Prior to the addition of fresh medium, the cells underwent double washing with JC‐1 staining buffer. Finally, the fluorescence intensities were measured for both JC‐1 monomers and aggregates using a plate reader (BioTek) set to excitation wavelengths of 490 and 525 nm, and corresponding emission wavelengths of 530 and 590 nm. To evaluate MMP changes, the fluorescence intensity ratio between the aggregated and monomeric forms of JC‐1 was calculated.

### 2.11 Western blotting assay

The Western blotting procedure was carried out according to the methods outlined in previous descriptions (Chen & Meng, 2022). Briefly, HUVECs were initially seeded and grown to confluence in 6‐well plates. Subsequently, they were exposed to distinct concentrations of TFEL or specific inhibitors (ML385 and LY294002) for a duration of 2 h, which was followed by MGO stimulation for 24 h. To prepare the cell lysates, the RIPA lysis solution (Beyotime Biotechnology) was utilized. Equal protein quantities from each experimental group were then subjected to SDS‐PAGE for protein separation and subsequently transferred onto PVDF membranes. The membranes were then incubated with antibodies according to the standard Western blotting protocol. The experiment involved the use of primary antibodies including anti‐Bax, anti‐Bcl‐2, anti‐Nrf2, anti‐HO‐1, anti‐phospho‐Akt^ser473^, anti‐AKT, and anti‐GAPDH. These antibodies were obtained from Cell Signaling Technology and used at specified working dilutions (1:1000). Horseradish peroxidase‐labeled goat anti‐rabbit IgG (work dilution 1:1000) was the secondary antibody employed, which was procured from Cell Signaling Technology (7074).

### 2.12 Determination of serum MGO levels

Upon experiment termination, blood samples were acquired from the mice under anesthesia. Subsequently, serum was derived through centrifugation at 2500 *g* and 4°C for 15 min. Serum MGO levels were determined using a commercial ELISA kit (Abcam, Cambridge, MA, USA) while following the provided protocol.

### 2.13 Histopathological analysis

After anesthetizing the mice, the aorta was surgically excised and rinsed thoroughly three times with physiological saline. Subsequently, the aortic tissue was immersed in formalin solution for a duration of 24 h to ensure proper fixation. Following this, the aorta underwent a gradual dehydration process using an ethanol gradient series and was then embedded in paraffin for histological sectioning. The tissue sections were stained with hematoxylin to visualize the nuclei and eosin to highlight the cytoplasm. After dehydration and sealing of the slides, the aortic sections were examined under microscope (Olympus BX53). The intima‐media thickness was precisely measured using ImageJ software, version 1.8.0 (National Institutes of Health, Bethesda, MD, USA).

### 2.14 TUNEL staining

To evaluate apoptosis in the mouse aorta, the TUNEL Assay Kit was utilized according to the manufacturer's guidelines (Beyotime Biotechnology). In brief, after anesthetizing the mice, the aorta was isolated, preserved in formalin for 24 h, and subsequently embedded in paraffin. The sections were then subjected to dewaxing and treated with DNase‐free Proteinase K (Beyotime Biotechnology) for 20 min at ambient temperature. Twice washing with PBS followed, and then the sections were incubated with the TUNEL assay solution at 37°C for 60 min in a dark environment. Finally, the sections were examined under a fluorescence microscope (Olympus) to observe the apoptotic cells.

### 2.15 Statistical analysis

All data are expressed as mean ± standard deviation (SD). For comparative analysis, we utilized the unpaired *t*‐test to assess differences between two groups, while one‐way ANOVA was employed for comparisons involving more than two groups. Statistically significant results were determined at a *p* value less than .05. All statistical analyses were carried out using GraphPad Prism version 8.0 (GraphPad Software, Boston, MA, USA).

## 3 RESULTS

### 3.1 Chemical profile analysis of TFEL

**FIGURE 1:**
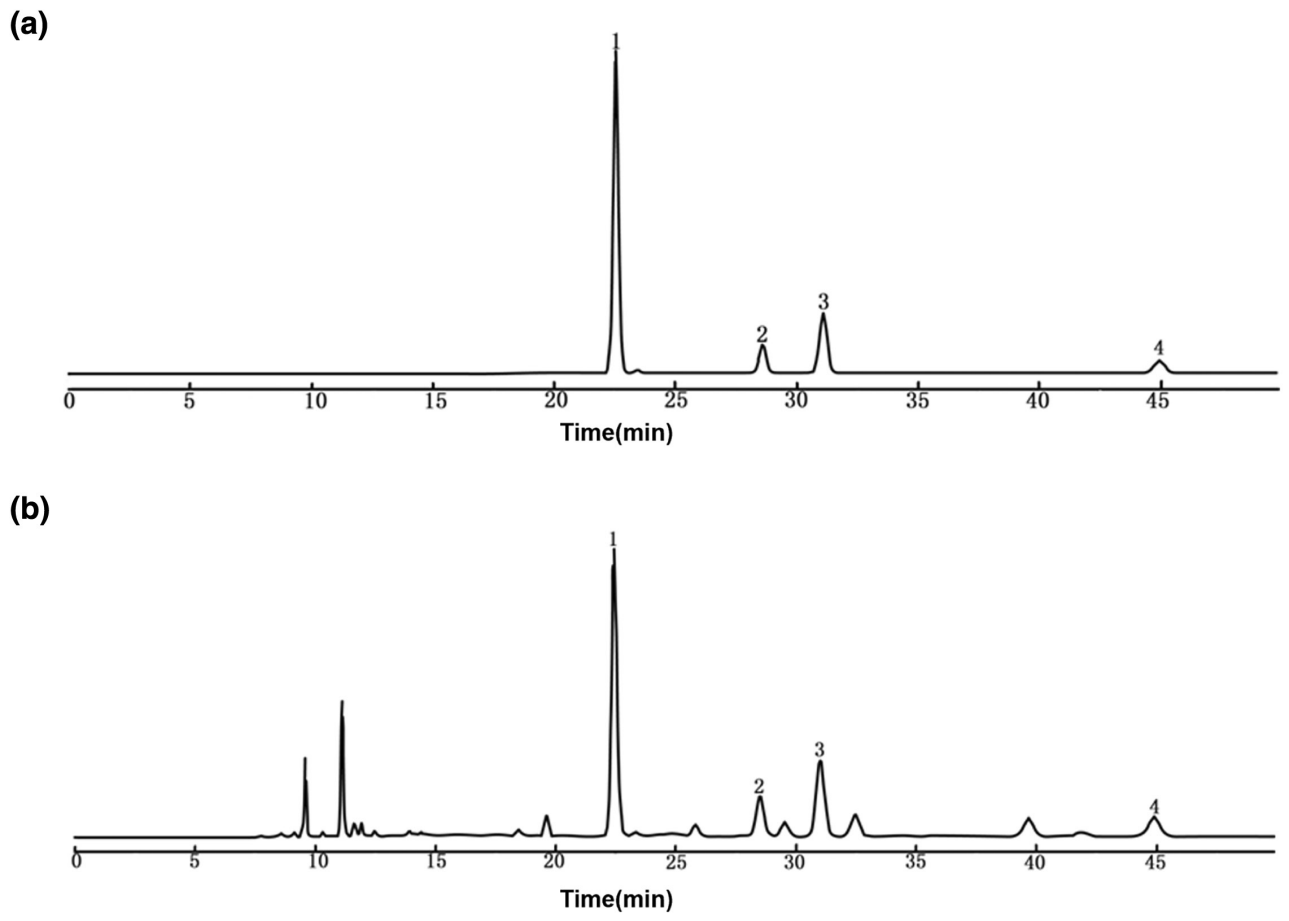
HPLC chromatograms of TFEL. (a) HPLC chromatogram of mixed standard in the standard solution. (b) HPLC chromatogram of TFEL. Peak (1) quercetin‐3‐*O*‐β‐d‐glucopyranosyl‐(1 → 2)‐α‐l‐arabinopyranoside, Peak (2) rutin, Peak (3) isoquercitrin, Peak (4) astragalin.

The chemical constituents comprising TFEL were examined via HPLC analysis. Based on their migration time using known chemicals, four main peaks were characterized as quercetin‐3‐*O*‐β‐d‐glucopyranosyl‐(1 → 2)‐α‐l‐arabinopyranoside (peak 1), rutin (peak 2), isoquercitrin (peak 3), and astragalin (peak 4) (Figure 1). The contents of the four main components of TFEL are shown in Table 1.

**TABLE 1:** HPLC analysis of TFEL.

Identified compounds	Retention time (min)	Peak area (mV)	Content (mg/g)
Quercetin‐3‐*O*‐β‐d‐glucopyranosyl‐(1 → 2)‐α‐l‐arabinopyranoside	22.39 ± 0.06	157,532.52 ± 5076.45	12.83 ± 0.66
Rutin	28.37 ± 0.05	41,536.91 ± 1951.04	3.50 ± 0.18
Isoquercitrin	30.94 ± 0.06	92,036.98 ± 3737.49	7.96 ± 0.35
Astragalin	44.90 ± 0.07	43,374.14 ± 1802.03	4.03 ± 0.19

*Note*: The values are expressed as the mean ± SD (*n* = 3).

### 3.2 TFEL reduces MGO‐induced cytotoxicity of HUVECs

**FIGURE 2:**
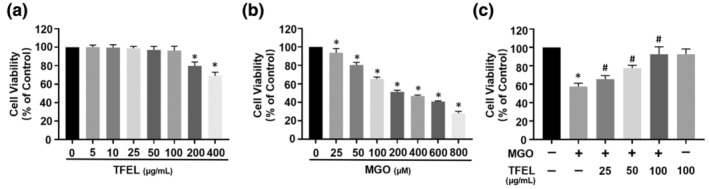
Effects of TFEL on MGO‐induced HUVECs cytotoxicity. (a) Effects of TFEL addition to HUVECs viability. HUVECs were treated with the indicated TFEL concentrations for 24 h prior to the assay. (b) The effect of different concentrations of MGO on the viability of HUVEC cells. HUVECs were treated with the indicated MGO levels for 24 h. (c) Effects of TFEL on MGO‐induced HUVECs cell viability. HUVECs were pretreated with different concentrations of TFEL (25, 50, and 100 μg/mL) and then exposed to MGO (200 μM) for 24 h. Data are presented as the mean ± SD (*n* = 6). *p < .05 versus Control, ^#^ *p* < .05 versus MGO.

We initially identified the limits of TFEL toxicity for HUVECs that were evaluated using cell viability assays. TFEL levels <200 μg/mL did not significantly affect HUVEC viability (Figure 2a). Based on these results, we chose concentrations of 25, 50, and 100 μg/mL to be used in future experiments. Similarly, MGO toxicity was assessed at 25–800 μM as previously reported (Wang et al., 2022). We found that HUVECs viability was negatively correlated with MGO concentration (Figure 2b). Particularly, MGO added at 200 μM produced a viability decline to 51.23 ± 2.53% compared to the control. This concentration was chosen for the subsequent experiments because it decreased cell viability by almost 50%. The in vitro experimental system involved pretreating HUVECs with TFEL at concentrations of 25, 50, and 100 μg/mL for 2 h prior to stimulating them with 200 μM MGO for a duration of 24 h. TFEL preincubation of the cells inhibited the MGO‐induced decrease in cell viability (Figure 2c). These data indicated a protective effect of TFEL.

### 3.3 TFEL mitigates apoptosis in HUVECs triggered by MGO

**FIGURE 3:**
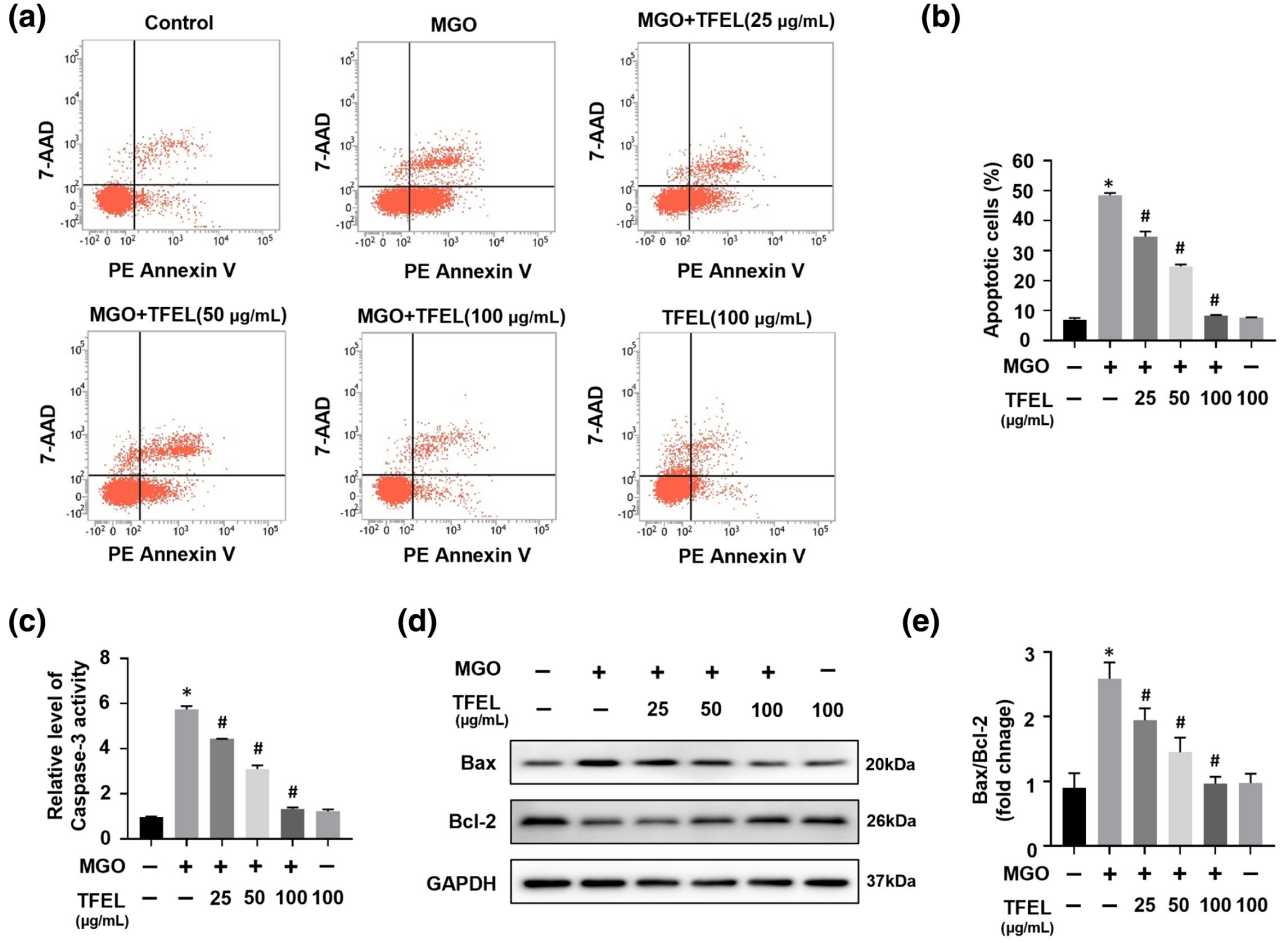
Effects of TFEL on MGO‐induced apoptosis in HUVECs. HUVECs were seeded in 6‐well plates and then pretreated with TFEL (25, 50, and 100 μg/mL) for 2 h followed by MGO (200 μM) stimulation for 24 h. (a, b) Flow cytometric analysis of HUVECs apoptosis. (c) The level of Caspase‐3 activity. (d, e) The effects of TFEL on MGO‐induced changes in the expression of apoptosis‐related proteins. Bax and Bcl‐2 were measured by Western blotting. The Bax/Bcl‐2 ratio was quantified by densitometry using GAPDH as a reference. Data are presented as the mean ± SD (*n* = 3). *p < .05 versus Control, ^#^ *p* < .05 versus MGO.

Given the association between reduced cellular viability caused by MGO and apoptotic cell death, we investigated the potential anti‐apoptotic protective effect of TFEL using an in vitro experimental system. The data revealed that TFEL pretreatment, particularly at 100 μg/mL, protected cells from the apoptotic effect caused by MGO (Figure 3a,b). Moreover, TFEL pretreatment attenuated the MGO‐induced caspase‐3 activity elevation (Figure 3c). The stimulation of MGO resulted in a substantial upregulation of the pro‐apoptotic BAX protein relative to the anti‐apoptotic Bcl‐2 protein, leading to an increased Bax/Bcl‐2 ratio. In contrast, TFEL intervention effectively restored the Bax/Bcl‐2 ratio to a normal level (Figure 3d,e). These findings indicated that TFEL prevented HUVECs apoptosis induced by MGO.

### 3.4 TFEL reduces MGO‐induced oxidative stress and restores MMP

**FIGURE 4:**
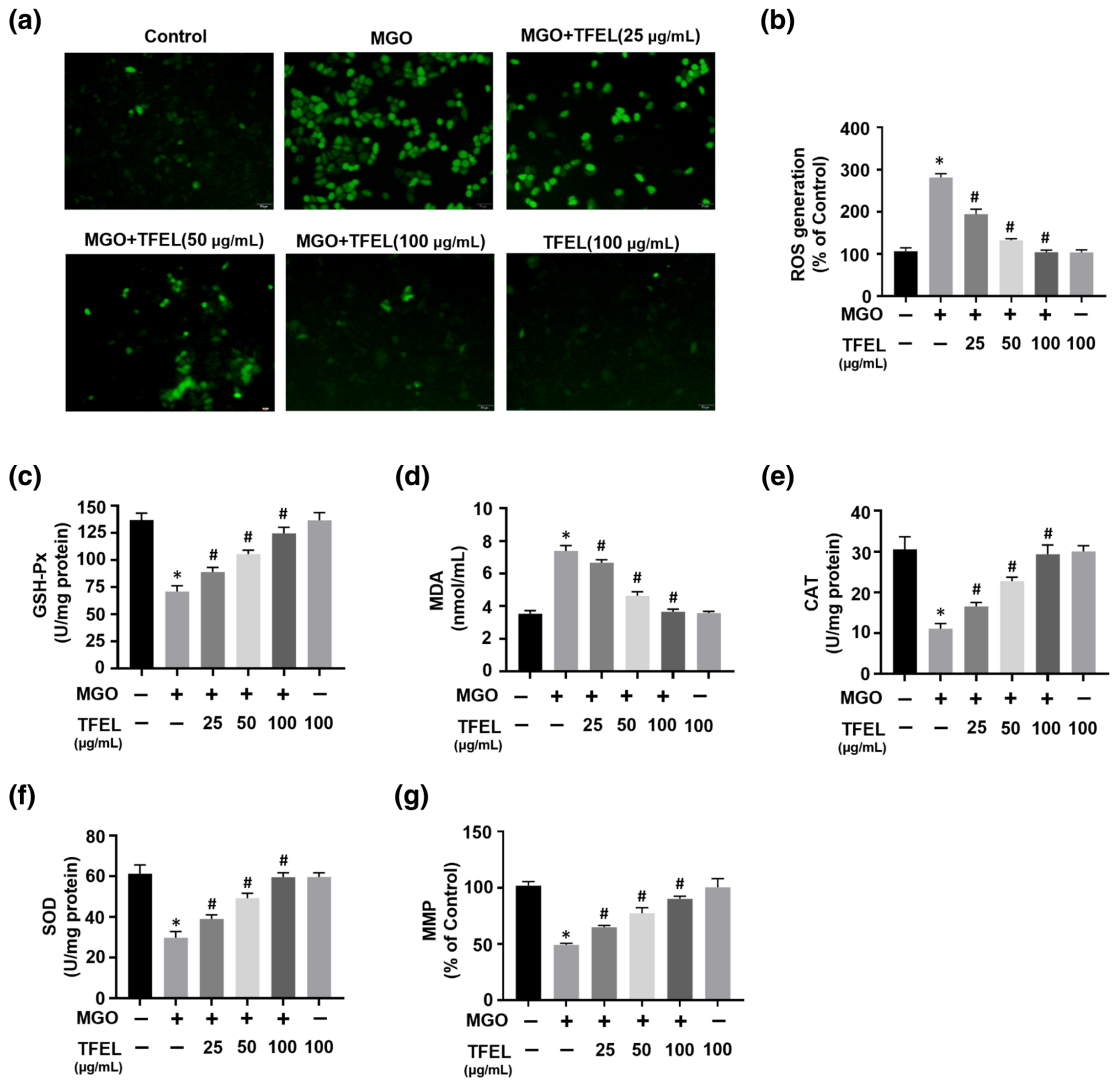
Effects of TFEL on MGO‐induced oxidative stress and MMP loss in HUVECs. Cells were pretreated with TFEL (25, 50, and 100 μg/mL) for 2 h followed by MGO (200 μM) exposure for 1 h. (a, b) Intracellular ROS levels following staining with a DCFH‐DA probe for 30 min. Representative micrographs are illustrated. Fluorescence intensity was recorded at the wavelength (488/525 nm) with a microplate reader. Magnification, 200×. Bar, 50 μm. (c–f) Cellular GSH‐Px, CAT, SOD activity levels, and MDA content. (g) JC‐1 probe assay to detect intracellular MMP changes. JC‐1 monomers (490/530 nm) and JC‐1 aggregates (525/590 nm) were measured with a microplate reader and the ratio of monomers to aggregates was calculated. Data are presented as the mean ± SD (*n* = 3). *p < .05 versus Control, ^#^ *p* < .05 versus MGO.

Excessive ROS generation and MMP loss are typical effects of oxidative stress injury. We, therefore, examined whether MGO addition to HUVECs resulted in elevated intrasellar ROS using the fluorescent probe DCFH‐DA. HUVECs exposure to MGO resulted in significant elevations in intracellular ROS. Interestingly, excess ROS were not detected in cells pre‐incubated with TFEL, particularly at 100 μg/mL (Figure 4a,b). The addition of MGO also significantly reduced the levels of the major components of antioxidant defense system, namely GSH‐Px, CAT, and SOD activities, while concurrently causing a notable elevation in MDA levels compared to the control group (Figure 4c–f). In contrast, preincubation with TFEL prevented GSH‐Px, CAT, and SOD decline as well as the MDA increase caused by MGO (Figure 4c–f). Decreased MMP predicts mitochondrial dysfunction and is involved in the process of apoptosis. Our data show that the addition of TFEL rescued the reduction of HUVECs MMP caused by MGO (Figure 4g). The findings of the study demonstrated that TFEL drastically reduced oxidative stress caused by MGO in HUVECs.

### 3.5 TFEL upregulates AKT phosphorylation and Nrf2/HO‐1 in MGO‐treated endothelial cells

**FIGURE 5:**
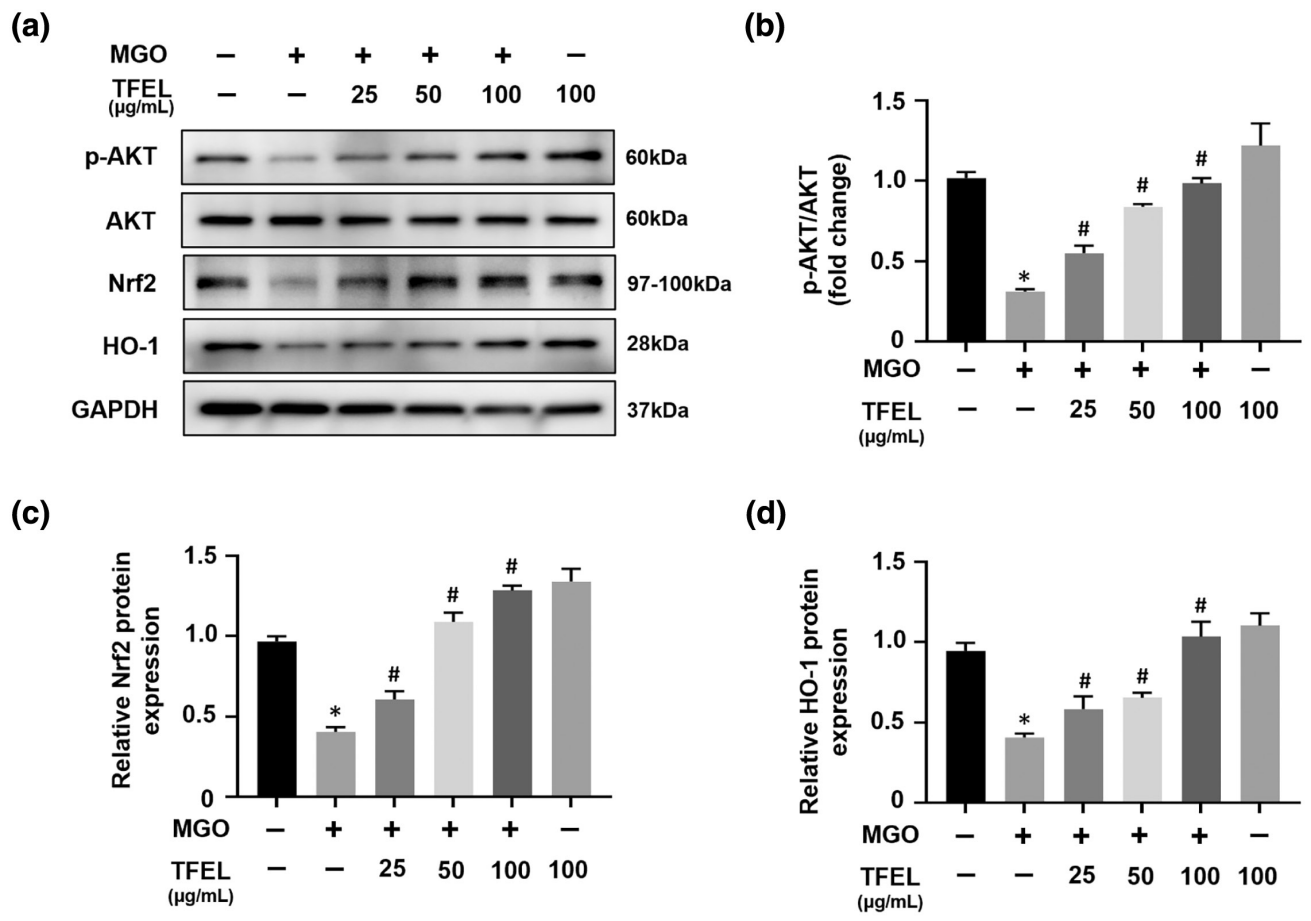
Effects of TFEL on MGO‐induced alterations in AKT phosphorylation, Nrf2, and HO‐1 levels in HUVECs. HUVECs were pretreated with TFEL for 2 h, followed by Western blotting for p‐AKT and AKT after 1 h of MGO treatment or Nrf2 and HO‐1 after 24 h of MGO treatment. (a) Representative Western blot bands depicting expression levels of p‐AKT, AKT, Nrf2, and HO‐1 in HUVECs for the indicated treatment groups. Protein expression levels of p‐AKT (b), Nrf2 (c), and HO‐1 (d) analyzed by densitometry of corresponding bands from the Western blot images. AKT or GAPDH were used as reference, respectively. Data are presented as the mean ± SD (*n* = 3). *p < .05 versus Control, ^#^ *p* < .05 versus MGO.

Considering TFEL's effectiveness in protecting against oxidative stress injury, we investigated if it could mitigate oxidative stress via regulation of the p‐Akt and Nrf2/HO‐1 – crucial mediators of the adaptive response to oxidative stress (Li et al., 2018). Our prior research revealed an association between MGO‐triggered AKT dephosphorylation and HUVECs apoptosis. Specifically, 30 min of MGO exposure significantly decreased p‐Akt levels, reaching a nadir at 60 min (Pang et al., 2017). Here we observed that MGO stimulation led to a notable reduction in the levels of Nrf2, HO‐1, and p‐Akt but the addition of TFEL prevented these alterations (Figure 5a–d). Together, the presented data implicate the involvement of p‐AKT and Nrf2/HO‐1 signaling cascades in MGO‐triggered apoptosis in HUVECs. TFEL may therefore act to prevent MGO‐induced apoptosis by upregulating these signaling pathways.

### 3.6 Nrf2's pivotal function in TFEL's defense against MGO‐triggered oxidative stress and apoptosis

**FIGURE 6:**
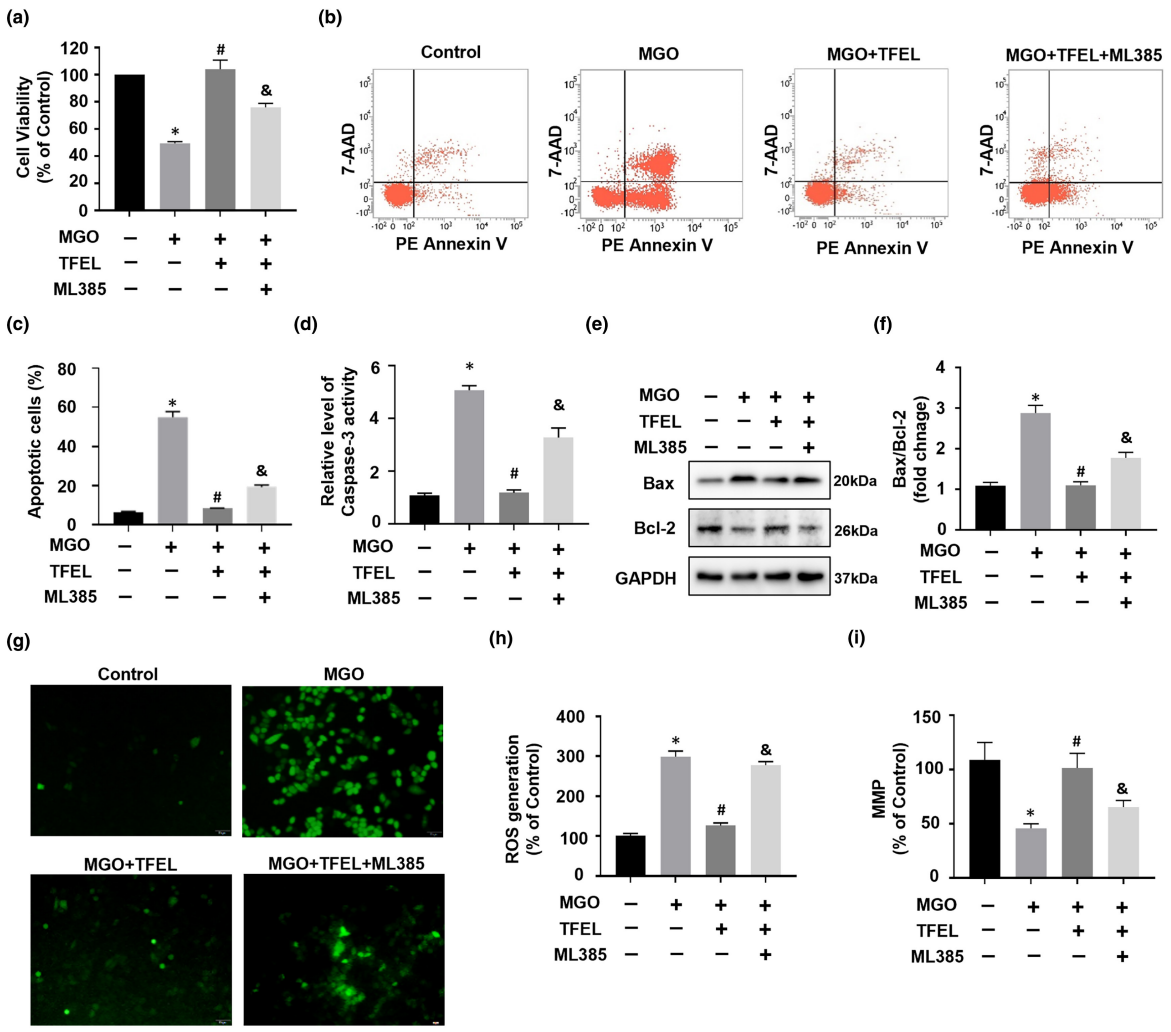
A Nrf2 inhibitor attenuates the protective effect of TFEL against MGO‐induced apoptosis and oxidative stress. HUVECs were pretreated with TFEL (100 μg/mL) for 2 h with or without ML385 (20 mM) before being exposed to MGO (200 μM) for 24 h. Cell viability (a) and flow cytometric analysis (b, c) of HUVECs apoptosis. Caspase‐3 activity (d) and protein expression of apoptosis‐related markers BAX and Bcl‐2 (e, f) assessed by densitometry of Western blots using GAPDH as a reference. (g, h) Intracellular ROS levels in HUVECs determined using a DCFH‐DA probe. (i) MMP of HUVECs assessed using the JC‐1 probe. Data are presented as the mean ± SD (*n* = 3). *p < .05 versus Control, ^#^ *p* < .05 versus MGO, ^&^ *p* < .05 versus MGO + TFEL.

Previous studies had indicated that the ROS scavenger *n*‐acetyl cysteine (NAC) was able to decrease MGO‐induced apoptosis in HUVECs, implying the involvement of oxidative stress signaling in the regulation of endothelial cell apoptosis. Hence, our attention centered on the Nrf2/HO‐1 signaling cascade, which plays a pivotal role in regulating oxidative stress. We utilized an Nrf2‐specific inhibitor, ML385, to investigate whether it modulated TFEL's defense against apoptosis induced by MGO in HUVECs. First, our results showed that ML385 addition to cells compromised TFEL's ability to counteract the decrease in cell viability caused by MGO (Figure 6a). Flow cytometric analysis with PE Annexin V and 7‐AAD revealed a similar trend in which ML385 partially eliminated the TFEL protective effect against MGO‐induced apoptosis (Figure 6b,c). Consistent with these results, TFEL pretreatment reduced caspase‐3 activity and the Bax/Bcl2 ratio relative to MGO treatment alone. Adding ML385 partially eliminated the inhibitory effect of TFEL on these apoptosis indicators (Figure 6d–f) and intracellular ROS generation (Figure 6g,h). Furthermore, the protective effect of TFEL against MGO‐induced MMP loss was inhibited by ML385 (Figure 6i). These findings reveal the importance of the Nrf2‐mediated signaling cascade in conferring protection against apoptosis and oxidative stress induced by MGO in endothelial cells. Moreover, the protective effects of TFEL on cell viability and antioxidant activities are tightly linked to Nrf2 signaling.

### 3.7 Inhibiting p‐Akt diminishes TFEL's anti‐apoptotic and anti‐oxidative effects against MGO

**FIGURE 7:**
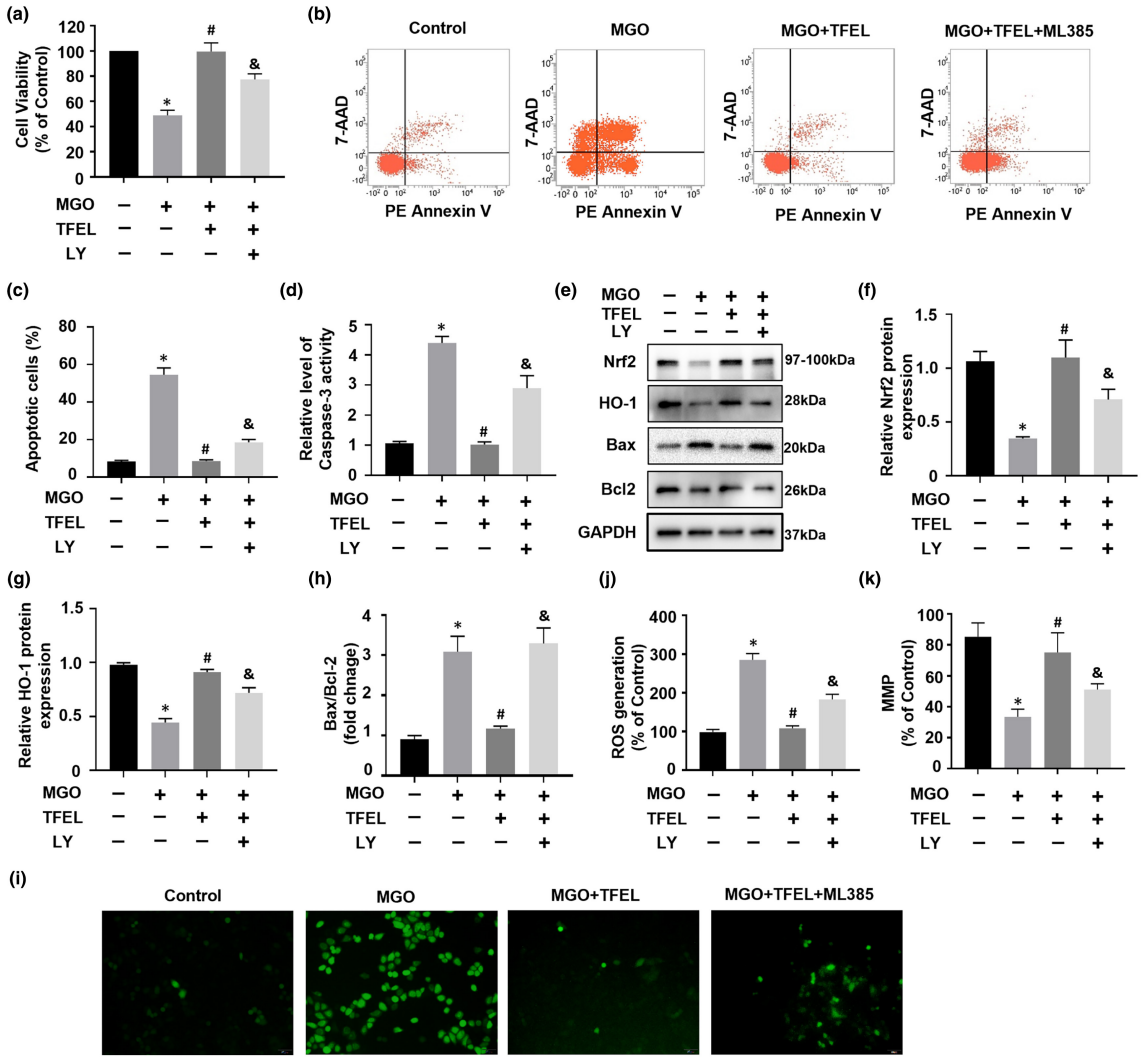
AKT inhibitor attenuates the upregulation of Nrf2/HO‐1 and decreases the protective effects of TFEL against MGO‐induced apoptosis and oxidative stress in HUVECs. Cells were pretreated with TFEL (100 μg/mL) for 2 h with or without LY294002 (50 μM) prior to MGO (200 μM) exposure for 24 h. Cell viability (a) and apoptosis (b, c) detected by flow cytometry. (d) The level of Caspase‐3 activity. Western blotting to quantify protein levels of Nrf2 (e), HO‐1 (f), BAX (g), and Bcl‐2 (h) using GAPDH as a reference. (i, j) Intracellular ROS levels in HUVECs detected with the DCFH‐DA probe. Representative fluorescence micrographs are shown Cellular MMP levels (k) detected using the JC‐1 probe. Fluorescence intensities of JC‐1 monomers (490/530 nm) and JC‐1 aggregates (525/590 nm) were measured and their ratios were calculated. Data are presented as the mean ± SD (*n* = 3). *p < .05 versus Control, ^#^ *p* < .05 versus MGO, ^&^ *p* < .05 versus MGO + TFEL.

Earlier investigations have identified Nrf2 as a downstream target of p‐Akt, prompting us to evaluate the impact of LY204002 (a PI3K inhibitor, which can inhibit p‐Akt level), on TFEL's protective effects against reductions in MGO‐induced cell viability in HUVECs. Pretreatment of HUVECs with LY204002 and TFEL for 2 h in the experimental system resulted in decreased cell viability compared with cells not exposed to LY204002 (Figure 7a). The inclusion of LY204002 in the experimental system significantly attenuated TFEL's ability to protect against apoptosis triggered by MGO (Figure 7b,c). Additionally, when compared to the TFEL and MGO combination, LY204002 showed a noteworthy upregulation in BAX/Bcl‐2 ratio along with an elevation in caspase‐3 activity (Figure 7d,e,h). Furthermore, Western blotting revealed that LY204002 significantly attenuated the TFEL‐induced upregulation of Nrf2 and HO‐1 expression (Figure 7e–g). The decreases in intracellular ROS levels due to TFEL addition were reversed by LY204002 addition, indicating that this drug significantly reduced the capacity of TFEL to inhibit ROS generation caused by MGO (Figure 7i,j). Additionally, LY204002 inhibited the ability of TFEL to protect HUVECs from MMP loss (Figure 7k). These results indicated that TFEL's protection of endothelial cells from MGO‐triggered apoptosis and oxidative stress requires the upregulation of Akt phosphorylation to regulate the Nrf2/HO‐1 pathway.

### 3.8 TFEL modulates physiology, oxidative stress, and aortic apoptosis in MGO injury model

**FIGURE 8:**
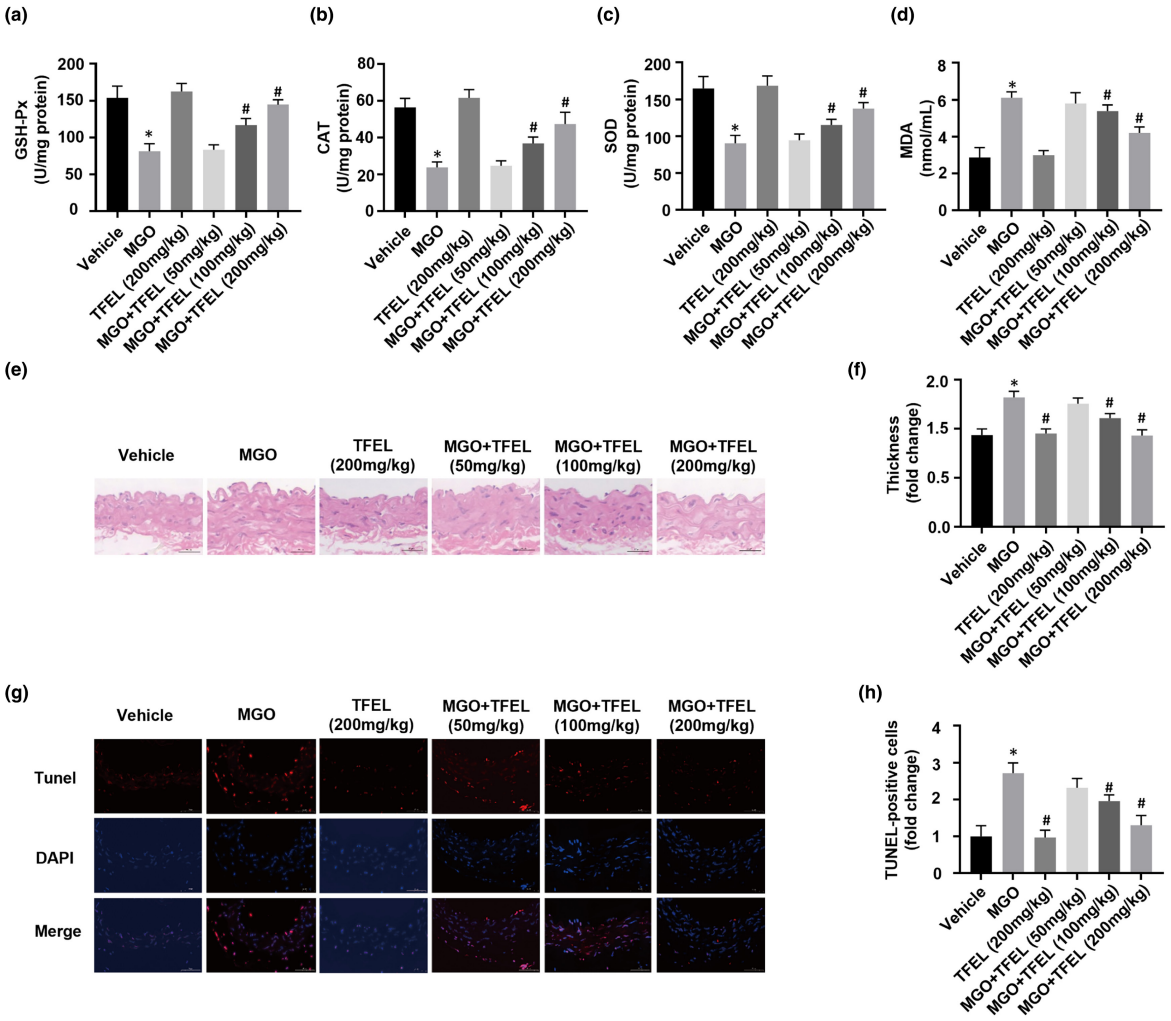
Effects of TFEL on oxidative stress status and aortic apoptosis using the murine model of MGO injury in C57BL/6 mice. Enzyme activity measurements of GSH‐PX (a), CAT (b), SOD (c), and MDA (d) content in serum. (e) Observation of pathological changes in aortic tissue under optical microscope: H&E. 80×. (f) The thickness of the intima‐media of the aorta. (g) TUNEL staining of sectioned mouse aortas from the indicated groups. Apoptotic cells stained red. Nuclear staining in blue (Hoechst 33258). 100×. (h) TUNEL‐positive cell numbers in the aortic vessel walls. Data are presented as the mean ± SD (*n* = 6). *p < .05 versus Vehicle, ^#^ *p* < .05 versus MGO.

To further investigate the potential of TFEL in modulating oxidative stress and protecting against apoptosis in a physiological context, we extended our experiments to an in vivo model. Over a period of 7 weeks, mice received either MGO, TFEL, or a combination of both. Body weight, daily food intake, fasting, and feeding blood glucose levels were recorded for the mice both at the experiment's outset and upon its conclusion. In the 7th week of the experiment, blood and aortic samples were collected from the mice. No noteworthy variations were observed in body weight, daily food intake, or fasting/feeding blood glucose levels among the vehicle group and the groups receiving either MGO, TFEL, or their combination (Table 2). Compared to the vehicle group, the MGO‐injected group exhibited markedly elevated serum MGO concentrations. However, upon administration of TFEL, particularly in the 100 and 200 mg/kg dosage groups, there was a notable decrease in serum MGO levels (Table 2). Serum GSH‐Px, CAT, and SOD activities were significantly lower and the MDA content was significantly higher for the MGO group relative to the vehicle group. However, the administration of TFEL (dose: 100 and 200 mg/kg) effectively counteracted the decrease in serum GSH‐Px, CAT, and SOD activities and the increase in MDA content caused by MGO exposure (Figure 8a–d). Additionally, H&E staining analysis of the aorta revealed that TFEL at doses of 100 and 200 mg/kg could inhibit the increase in aortic thickness stimulated by MGO (Figure 8e,f). TUNEL staining of aortic vessels indicated that MGO‐induced apoptosis was significantly suppressed after TFEL administration (100 and 200 mg/kg) (Figure 8g,h). These findings indicated that TFEL ameliorates oxidative stress in vivo and exhibits a protective function toward the aorta by counteracting MGO‐triggered apoptosis.

**TABLE 2:** Effects of TFEL on physiological characteristics and glycometabolism in the MGO injury model.

Group	Time point	Vehicle	MGO	TFEL (200 mg/kg)	MGO + TFEL (50 mg/kg)	MGO + TFEL (100 mg/kg)	MGO + TFEL (200 mg/kg)
Body weight (g)	Before the intervention	22.175 ± 0.89	21.74 ± 0.40	21.96 ± 0.62	21.35 ± 0.51	21.81 ± 0.33	22.13 ± 0.82
	After the intervention	24.25 ± 1.36	25.175 ± 1.27	24.74 ± 0.99	24.64 ± 0.92	24.79 ± 1.28	24.33 ± 1.28
Food intake (g/day/mouse)	Before the intervention	4.03 ± 0.17	4.01 ± 0.18	4.06 ± 0.16	4.05 ± 0.16	4.00 ± 0.29	4.03 ± 0.16
	After the intervention	4.04 ± 0.26	4.11 ± 0.16	4.00 ± 0.13	4.01 ± 0.25	3.98 ± 0.26	3.99 ± 0.18
Fasting glucose (mmol/L)	Before the intervention	4.81 ± 0.73	5.00 ± 0.60	5.01 ± 0.75	4.99 ± 0.38	4.94 ± 0.24	5.08 ± 0.47
	After the intervention	4.86 ± 0.81	5.06 ± 0.60	4.96 ± 0.69	5.06 ± 0.30	4.99 ± 0.25	4.91 ± 0.50
Fed glucose (mmol/L)	Before the intervention	8.75 ± 1.0	8.51 ± 1.01	8.62 ± 1.23	8.49 ± 1.15	8.78 ± 0.98	8.69 ± 1.28
	After the intervention	8.44 ± 0.76	8.56 ± 0.96	8.67 ± 1.15	8.55 ± 1.15	8.86 ± 0.93	8.76 ± 0.81
MGO (ng/mL)	After the intervention	20.07 ± 2.71	79.10 ± 8.31*	19.94 ± 2.23	77.39 ± 6.86	59.66 ± 5.08^#^	40.28 ± 7.56^#^

*Note*: Data are presented as the mean ± SD (*n* = 6).

*p < .05 versus Vehicle, ^#^ *p* < .05 versus MGO.

## 4 DISCUSSION

Endothelial cells, which compose a solitary layer lining the blood vessels, play a critical role in stable and normal vascular homeostasis (Michiels, 2003; Trimm & Red‐Horse, 2023). Endothelial cell apoptosis, which disrupts vascular homeostasis and contributes to vascular barrier dysfunctions, vasoconstriction and dilation, inflammatory responses, and thrombosis, serves as the pathological basis for diabetic vascular complications (Trimm & Red‐Horse, 2023). Therefore, the identification of novel therapeutic candidates and understanding of the mechanisms involved in diabetes‐related endothelial cell apoptosis remain a focus of current research. Recent studies have highlighted the potential of plant‐derived natural products in inhibiting endothelial cell apoptosis. For example, Thai green papaya salad (Som Tam) containing unripe Carica papaya can significantly reduce endothelial cell apoptosis and inflammation (Jarisarapurin et al., 2021). Natural products including resveratrol derivative pterostilbene can protect endothelial cells from MGO‐induced cytotoxicity through the modulation of glyoxalase activity, reduction of oxidative stress levels, and regulation of apoptotic processes (Tang et al., 2021). According to the present data, it is demonstrated that TFEL performs similar protective functions for endothelial cells.

Methylglyoxal is primarily formed from glycolysis byproducts and hyperglycemia is an essential factor for its formation and accumulation (Matafome et al., 2013; Schalkwijk & Stehouwer, 2020). Critical limb ischemia patients' plasma MGO levels have been found to correlate strongly with amputation and mortality rates, indicating a potential key role of MGO in vascular complications related to diabetes (Hanssen et al., 2021). MGO triggers endothelial cell inflammation, oxidative stress, and apoptosis. It has been widely used in the construction of diabetes‐related endothelial injury models (Jarisarapurin et al., 2021; Wang et al., 2022). Our data demonstrated that exposing HUVECs to MGO considerably diminished their viability and increased apoptotic rates. However, pre‐treatment with TFEL enhanced cell viability and protected endothelial cells from apoptosis induced by MGO. TFEL's ability to protect against apoptosis was further corroborated in vivo, utilizing an MGO‐induced vascular injury model. In this model, TFEL effectively suppressed MGO‐triggered apoptosis and aortic thickening. *E. ulmoides* exhibits similar chemical compositions in its bark and leaves. Previous studies have demonstrated that bark flavonoids exert a protective effect against LPS‐induced IPEC‐J2 cell apoptosis (Hussain et al., 2020; Shi et al., 2022). Our results show similar findings that TFEL prevented apoptosis in MGO‐induced endothelial cells, suggesting its potential as a candidate for mitigating diabetic vasculopathy. Towards these ends, the current study indicated that the TFEL protective effects are likely achieved via the Nrf2‐mediated signaling cascade.

Due to altered intracellular lipid peroxidation levels and diminished antioxidant enzyme activity, the antioxidant system becomes destabilized, ultimately inducing oxidative stress. Thus, elevated ROS are a dominant factor in endothelial cell injury and can initiate cardiovascular disease (Incalza et al., 2018). Furthermore, MGO‐induced ROS generation and destabilization of the antioxidant system are responsible for endothelial cell apoptosis in the mouse model (Jarisarapurin et al., 2021; Tang et al., 2021). Elevated levels of ROS have been implicated in inducing oxidative stress, which subsequently causes damage to cellular macromolecules, including proteins, lipids, and even DNA (Ho et al., 2013; Juan et al., 2021). Dietary supplementation with *E. ulmoides* flavonoids can attenuate diquat‐induced intestinal damage in piglets by alleviating oxidative stress (Yuan et al., 2017). We therefore hypothesized that TFEL could protect endothelial cells from apoptosis by resisting oxidative stress. Our data confirmed the above hypothesis and TFEL significantly attenuated MGO‐induced oxidative stress in HUVECs in vitro by inhibiting ROS generation via stabilizing GSH‐Px, CAT, and SOD activities. Furthermore, TFEL dietary supplementation in the mouse model significantly reduced MDA and restored antioxidant enzyme levels of GSH‐Px, CAT, and SOD in serum, thus reducing MGO‐induced oxidative stress. Overall, TFEL demonstrates protective effects against apoptosis induced by MGO, both in vivo and in vitro, by suppressing excessive ROS production and activating the antioxidant defense system.

Excessive ROS generation decreases the MMP and increases membrane permeability resulting in mitochondria‐dependent apoptotic pathways (Gao et al., 2016). In general, decreased MMP is considered a hallmark of mitochondrial dysfunction (Sinha et al., 2013; Wang et al., 2022). MGO exposure decreases MMP, increases mitochondrial membrane permeability, and activates mitochondrial‐dependent apoptotic pathways. We found that MGO stimulation significantly reduced MMP in HUVECs while TFEL pretreatment inhibited MGO‐induced MMP loss. The BAX/Bcl‐2/caspase‐3 pathway is a key regulator of intrinsic apoptosis resulting from mitochondrial dysfunction (Bock & Tait, 2020). Therefore, the regulation of mitochondrial‐dependent apoptosis may be a possible mechanism for TFEL to protect against MGO‐induced HUVEC apoptosis. Our results confirm this hypothesis, demonstrating that TFEL inhibits caspase‐3 activity and suppresses the elevation of the BAX/Bcl‐2 ratio caused by MGO in vitro. Thus, our findings indicate TFEL's effectiveness in protecting HUVECs from apoptosis induced by MGO through the modulation of mitochondrial function and apoptosis signaling pathways.

Nrf2, a pivotal transcription factor, orchestrates the concerted activation of defense genes that encode for antioxidant proteins, which are triggered through their binding to genomic antioxidant response elements under oxidative stress conditions (Alam & Cook, 2003). Interestingly, artificially activating Nrf2 can alleviate endothelial injury induced by MGO (Wang et al., 2022). We found that TFEL significantly restored Nrf2 and HO‐1 protein levels that were reduced in HUVECs following MGO stimulation. This indicated that Nrf2 activation may be an essential pathway for TFEL's protective effects of TFEL in inhibiting oxidative stress and apoptosis. Numerous investigations have revealed a strong association between the activation of Nrf2/HO‐1 signaling pathway and the cytoprotective effects exerted by various natural monomers or extracts on endothelial cells (Liu et al., 2022; Zhang et al., 2021). The addition of *E. ulmoides* flavonoids, lignans, and extracts to cells also resulted in the enhanced activation of the Nrf2 signaling pathway, thereby reducing oxidative damage and exerting protective effects on the cells (Han et al., 2022; Kwon et al., 2016; Xiao et al., 2019). We directly confirmed a role for Nrf2 using the specific inhibitor ML385, which notably diminished the defensive actions of TFEL against oxidative damage, loss of MMP, and apoptosis induced by MGO. Altogether, the obtained findings indicated that TFEL protects against MGO‐induced HUVECs apoptosis by triggering Nrf2/HO‐1.

Our results have elucidated a role for TFEL in activation of Nrf2 signaling although the upstream regulatory mechanism remains unclear. AKT plays a crucial role in PI3K signaling and regulates cell apoptosis (Franke et al., 2003; Zhang et al., 2022). Previous studies have observed a reduction in AKT phosphorylation correlating with MGO‐induced endothelial cell apoptosis (Pang et al., 2017). In agreement with those observations, the present data indicate that TFEL restored AKT phosphorylation that was decreased by MGO addition in HUVECs, demonstrating that TFEL had activated AKT signaling. AKT phosphorylation can promote Nrf2 transcriptional activity, thereby conferring protection from apoptosis induced by oxidative stress (Dong et al., 2020; Li et al., 2018). Although the above data demonstrated TFEL activation of AKT signaling, it was unclear whether TFEL‐mediated Nrf2 pathway activation was directly regulated by AKT signaling. We therefore used the PI3K‐specific inhibitor LY294002 and confirmed the link between AKT and Nrf2 signaling pathways. An MGO‐induced endothelial cell injury model revealed that p‐Akt inhibition notably reduced the TFEL‐mediated upregulation of Nrf2 and HO‐1 protein levels. The findings revealed that TFEL‐mediated Nrf2/HO‐1 signaling pathway activation was regulated by p‐Akt. Furthermore, LY‐294002 abolished TFEL's protective functions in preventing apoptosis, damage to the antioxidant enzyme system, and loss of MMP in the MGO‐induced endothelial injury cell model. These findings further underscore the significant role played by TFEL in countering endothelial cell apoptosis induced by MGO, via the activation of the AKT‐Nrf2 signaling pathway. However, further in vivo investigations are warranted to verify the AKT/Nrf2 signaling pathway activation by TFEL and to elucidate any other possible pathways associated with TFEL's protective mechanisms against MGO‐induced apoptosis in endothelial cells.

## 5 CONCLUSION

**FIGURE 9:**
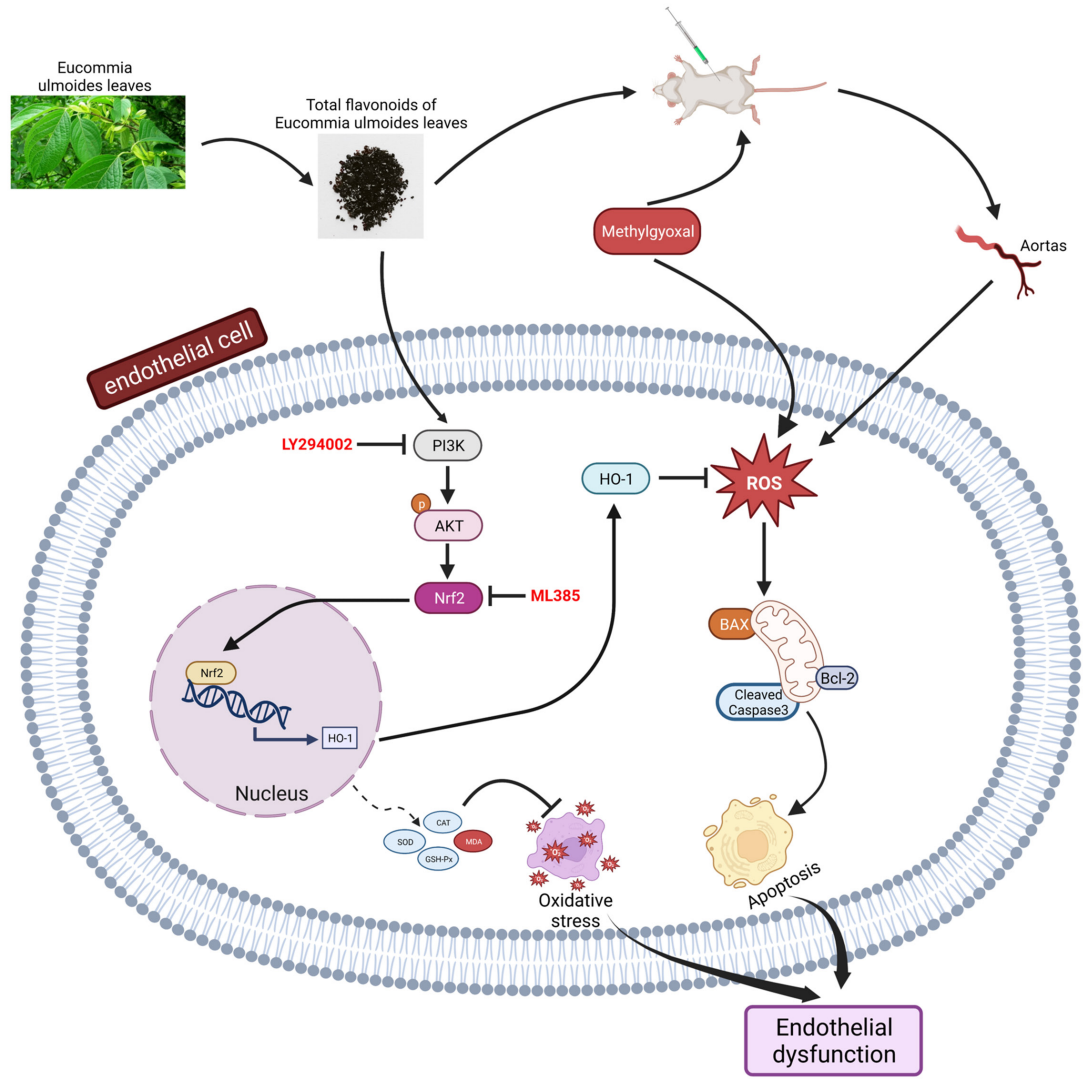
The molecular mechanism of TFEL protection against MGO‐induced endothelial injury.

Our experimental evidence, utilizing both in vivo and in vitro models, has shown that TFEL safeguards endothelial cells from MGO‐induced apoptosis by activating AKT/Nrf2 signaling and inhibiting the ROS‐induced apoptotic signaling pathway (Figure 9). These findings underscore the prospective utility of TFEL as an innovative therapeutic agent for enhancing endothelial function and managing vascular complications associated with diabetes.

## AUTHOR CONTRIBUTIONS

Xin Deng: Data curation (lead); conceptualization (equal); software (equal); methodology (equal); writing – original draft (equal). Qianfeng Wu: Investigation (equal); methodology (equal); validation (equal). Youping Liu: Funding acquisition (equal); supervision (equal); writing – review and editing (equal).

## ACKNOWLEDGEMENTS

The authors gratefully acknowledges the financial support provided by the Foundation of Luzhou Municipal Science and Technology Bureau (grant number: 2022‐JYJ‐144), Xinglin Scholar Research Promotion Project of Chengdu University of Traditional Chinese Medicine (grant number: CXTD2018011), and Scientific Research Project of Southwest Medical University (grant number: 2022ZD009).

## FUNDING INFORMATION

This work was supported by the Foundation of Luzhou Science and Technology and Talent Bureau (2022‐JYJ‐144), Xinglin Scholar Research Promotion Project of Chengdu University of Traditional Chinese Medicine under Grant (CXTD2018011), and Foundation of Southwest Medical University (2022ZD009).

## CONFLICT OF INTEREST STATEMENT

The authors declare that they have no competing interests.

## ETHICS STATEMENT

All animal studies were approved by the Experimental Animal Ethics Committee of Southwest Medical University (Approval No. 20221001‐006) and were conducted in strict accordance with the NIH Guide for the Care and Use of Laboratory Animals.

## Data Availability

The data supporting the results of this investigation can be accessed by contacting the corresponding author.
